# Old world versus new world: life-history alterations in a successful invader introduced across Europe

**DOI:** 10.1007/s00442-013-2776-7

**Published:** 2013-09-25

**Authors:** Michael G. Fox, Gordon H. Copp

**Affiliations:** 1 Environmental and Resource Studies Program and Department of Biology, Trent University, 1600 West Bank Drive, Peterborough, ON K9J 7B8 Canada; 2 Salmon and Freshwater Team, Centre for Environment, Fisheries and Aquaculture Science, Lowestoft, Suffolk, NR33 0HT UK; 3 Centre for Conservation Ecology and Environmental Science, Bournemouth University, Poole, Dorset, BH12 5BB UK; 4 Environmental and Life Sciences Graduate Program, Trent University, 1600 West Bank Drive, Peterborough, ON K9J 7B8 Canada

**Keywords:** Enemy release, Pumpkinseed, Somatic growth, Species introduction, Temperature

## Abstract

We examined differences in pumpkinseed (*Lepomis gibbosus*) life-history traits between native North American and introduced European populations, and tested three life-history predictions related to the effect of temperature, growth, waterbody size, and the presence/absence of predators on native and non-native populations. Pumpkinseed populations exhibit more ‘opportunistic’ traits (earlier maturity, smaller size at maturity, and higher reproductive allocation) in their introduced European range than those in their native range. Predictions of life-history traits were improved when indicators of juvenile growth rate (mean length at age 2), waterbody size (surface area), and thermal regime (air temperature degree-days above 10 °C) were incorporated into models along with continental location, but European pumpkinseed populations exhibit more opportunistic life-history traits than North American populations even when these factors are accounted for. Native pumpkinseed in waterbodies containing piscivores mature later and at a larger size, and have lower gonadosomatic indices than those in waterbodies lacking piscivores, whereas there is no significant difference in the same three life-history traits between European waterbodies containing or lacking piscivores. Because congeneric competitors of the pumpkinseed are absent from Europe, the apparent absence of a predator life-history effect there could also be due to the absence of the major sunfish competitors. In either case, the evolution and maintenance of more opportunistic traits in European pumpkinseed can likely be attributed to enemy release, and this may explain the successful establishment and spread of pumpkinseed in many parts of Europe.

**Electronic supplementary material:**

The online version of this article (doi:10.1007/s00442-013-2776-7) contains supplementary material, which is available to authorized users.

## Introduction

Life-history theory is particularly well suited for examining biological invasions because it can predict life-history traits that are adaptive to the abiotic and biotic environment where the new species has been introduced (see Duncan et al. 2003; Bøhn et al. 2004; García-Berthou 2007). Invasion success in areas outside a species’ native range depends upon a species’ ability to adapt to novel environments. Highly adaptable species are generally successful in a wide range of environments or across a broad geographical range, which may explain why ‘previous invasion history’ is amongst the best predictors of invasiveness (e.g., Kolar and Lodge 2001). Life-history traits exhibited by successful invaders may be the result of their ability to switch between alternative developmental trajectories (Kováč et al. 2009) and/or to exploit differences in community composition, which may include a release from native competitors, native diseases, and/or from native predators (Keane and Crawley 2002; Alpert 2006).

Successful invaders often exhibit a wide variation in life-history traits, with differences evident between native and non-native populations (e.g., MacInnis and Corkum 2000; Fox et al. 2007; Kováč et al. 2009), or with dynamic shifts occurring over the course of an invasion (e.g., Bøhn et al. 2004; Gutowsky and Fox 2012). Biological traits that would be useful during the early phase of an invasion may be different than those that would be useful at a later phase (see Kolar and Lodge 2002; García-Berthou 2007). Thus, while ‘*r*-selected’ life-history traits (MacArthur and Wilson 1967) may be favored during the initial stages of a species’ establishment and spread in a novel environment because of low density and, in many cases, high food availability per individual (Phillips et al. 2010), ‘*K*-selected’ life-history traits, like lower reproductive investment and greater investment in individual offspring, may be favored where density levels are high enough to produce a more competitive environment.

A freshwater species with a very successful history of establishment and invasion outside its native range is the pumpkinseed (*Lepomis gibbosus*). Native to eastern and central North America, it is one of the ten most introduced aquatic species worldwide, with one of the highest rates of successful establishment (García-Berthou et al. 2005). This species inhabits a range of waterbody types, and can survive under severe environmental conditions, including hypoxia and high temperature (Crivelli and Mestre 1988; Fox and Keast 1991; Farwell et al. 2007). The pumpkinseed was introduced into European waters during the late nineteenth century (Vivier 1951), and is now established in at least 28 European countries (Copp and Fox 2007). Non-native populations exist as far north as Norway (Cucherousset et al. 2009), but they are most invasive in southern Europe, including the Iberian Peninsula where their expansion has been rapid and concerns have been raised about their impact on endemic species (García-Berthou and Moreno-Amich 2000; Elvira 2001).

As a nest-guarding species, the pumpkinseed could be considered an ‘equilibrium’ life-history strategist under the classification system of Winemiller and Rose (1992), although it also exhibits some ‘opportunistic’ traits, such as batch spawning (Crivelli and Mestre 1988). In comparing populations at the northern and southern ends of their native and non-native ranges, Fox et al. (2007) demonstrated that the pumpkinseed exhibits the most opportunistic traits where it is considered most invasive, and suggested that the combination of equilibrium characteristics and an opportunistic response to the novel environment appears to be associated with the species’ invasion success in this region. They also noted the possibility that the observed life-history differences among the regions studied were due to regional differences in aquatic communities, such as the absence of competitors, predators, and parasites in successfully invaded areas outside the species’ native range (see Torchin et al. 2003; Alpert 2006).

In the present study, we investigate differences in pumpkinseed life-history traits between native North American and introduced European populations in the light of demographic and environmental differences on both continents that could potentially affect the evolution and/or phenotypic expression of these traits. In particular, we consider differences in temperature, size of waterbodies where pumpkinseed are found, and biotic characteristics such as juvenile growth rate and the occurrence of obligate piscivores as potential regulating factors. We tested the following hypotheses: (1) pumpkinseed life-history traits will differ between introduced European and native North American populations; (2) pumpkinseed life-history traits (age and size at maturity and reproductive allocation) will be related to the rate of juvenile growth, the thermal environment, and the size of the waterbody; and (3) the presence/absence of both piscine predators and lepomid competitors will affect life-history traits on both continents. We predicted that age and size at maturity would decrease and reproductive allocation would increase with temperature, given the effect of temperature on the physiological rate of development in ectotherms (Atkinson 1994; Van der Have and de Jong 1996), as well as the temperature-related predictions of life-history models (Berrigan and Charnov 1994; Gillooly et al. 2002; Kingsolver and Huey 2008). We further predicted that introduced populations would show more opportunistic life-history traits (earlier maturity at a smaller size and greater reproductive allocation) than native North American populations, given the absence of congeneric or conspecific pumpkinseed competitors as well as the dearth of native predators in Europe and the theoretical and empirical influence of predators on the life-history traits of prey fishes (e.g., delayed maturity and larger size at maturity to increase survival in the presence of gape-limited predators; Reznick and Endler 1982; Belk and Hales 1993; Rennie et al. 2010). Finally, to determine whether piscivory could be a major factor influencing life-history differences between native North American and non-native European populations, we tested the prediction that intercontinental differences in pumpkinseed life-history traits would not be manifested when comparing North American and European populations from waterbodies that contain obligate piscivores.

## Materials and methods

### Life-history database

The data used for our study were generated from a combination of previously published pumpkinseed population research, supplemented by life-history and growth data collected by one or both authors over the past 18 years (Table 1; Appendix 1). Pumpkinseed life-history data were available for 90 populations (46 North American, 44 European), excluding populations living in artificially heated waterbodies. The latter were not used because the artificially elevated temperature regime would affect the life-history traits of such populations (Sandström et al. 1995); Dembski et al. 2006), and because air temperature norms for weather stations in the vicinity of these sites would not be representative of the thermal environment of these waterbodies. The 90 populations in our database cover most of the north–south range of the pumpkinseed on both continents. Data from all but 7 of the populations were collected by research teams supervised by one or both of the co-authors, with pumpkinseeds sampled around the beginning of the breeding season, and growth and life-history data obtained using standardized procedures (see Fox 1994). Data for the 7 populations not collected by our research teams were available from published sources. In most cases, the data available were for a single year only, but means were used for each variable when more than 1 year of life-history data were available for a population.

**Table 1:** Life-history database of native North American (*n* = 46) and introduced European (*n* = 44) pumpkinseed (*Lepomis gibbosus*) populations by type of waterbody and presence/absence of obligate predators

Continent	Waterbody type				Obligate predators		
	Stream	Lake/reservoir	Pond	Other	Yes	No	Unknown
North America	7	36	3	–	40	6	–
Europe	9	9	22	4	28	15	1

Life-history variables included in the study were mean age and length at maturity and gonadosomatic index (GSI) of females. GSI is the relative mass of the ovary to the non-ovarian mass of the fish [GSI = 100 × ovary mass × (wet somatic mass − ovary mass)^−1^], and was used as an indicator of reproductive allocation. Mean age at maturity was calculated where possible from the proportion of mature females in each age-class, using a formula adapted from DeMaster (1978), and mean length at maturity was calculated from the same formula, substituting 10 mm total length (TL) size-classes for age-class (Trippel and Harvey 1987). Population means for these traits were calculated for females in order to avoid anomalies associated with alternative life-history strategies of males and because gonad mass of males does not represent most of the energy put into reproduction (Gross 1979).

Because the timing of maturity in pumpkinseeds is related to their growth rate (Fox 1994), mean length at age 2, an indicator of the pre-maturational growth rate, was included as a variable as it has been used as a juvenile growth indicator in several previous life-history studies of North American (Fox 1994) and European populations (Fox and Crivelli 2001; Villeneuve et al. 2005; Fox et al. 2007). For this variable, the population mean was calculated, where possible, by averaging the back-calculated mean length at age 2 of each year-class present in the sample, or, if the data were unavailable, the value was taken from the length at age estimate supplied by the data source. When length values in the original data sources were given in fork or standard length, these values were converted to TL using the formulae provided in Copp et al. (2002).

Waterbody surface area has been related to growth rates of fishes (e.g., Purchase et al. 2005), and given the relationship between growth and maturation, we included surface area as an independent variable in our assessment of life-history differences between North American and European pumpkinseed populations. Surface area data were available for most of our study lakes, ponds, and reservoirs from published and online sources. Surface area data were unavailable for fluvial sites and in any event, area estimated for lotic sites would not be comparable to that estimated for lentic sites because of the open nature of the former.

### Assessment of thermal environment

Water temperature data were unavailable for most of the waterbodies used in the database, and, while latitude provides a rough indicator of the relative thermal conditions of waterbodies within a region, it is unsuitable for comparisons of waterbodies across North America and Europe, where broad-scale climatic factors differ greatly. For these reasons, air temperature was used as an indicator of the thermal environment, and historical mean monthly temperatures from the meteorological station nearest to each waterbody were used to generate an estimate of degree-days for that site. Historical temperature data were taken from either the U.S. National Climatic Data Center or the Global Historical Climatology Network. Records were not always available for the same period, but in most cases we had a 29- to 37-year record available up to the year 1990. Monthly air temperature means were used to generate annual degree-day estimates by subtracting 10 °C from each mean monthly value for a given site, and summing the months with positive remainders. Estimates were based on the 10˚ minimum because this is the standard degree-day indicator closest to the temperature at which pumpkinseed cease feeding (8.5˚; Keast 1968), and because the same measure has been successfully used to evaluate differences in temperature–growth relationships in native and introduced smallmouth bass (*Micropterus dolomieu*), a centrarchid with a similar native range to that of the pumpkinseed (Dunlop and Shuter 2006). The relationship between latitude and degree-days (DD) was highly significant on both continents [Europe: DD = 8,152 – 146 × (latitude); North America: DD = 8,201 − 161 × (latitude); *r* ^2^ > 0.92, *P* < 0.001 in both cases], but at equivalent latitudes, North American sites tend to be 500–700 DD cooler than European sites.

### Classification of predator and competitor regimes

To assess the predation pressure to which each population was exposed, we used presence/absence of obligate piscivores capable of preying on juvenile and adult pumpkinseed. Indicators of predator abundance were not used because they were generally unavailable for the populations on our database, and, where such indicators were present, they were not collected in the same manner in different areas. The species considered as obligate piscivores for the purpose of this study were the largemouth bass (*Micropterus salmoides*), any of the large-bodied esocids (*Esox* *lucius*, *E*. *masquinongy*) as well as the walleye (*Sander* *vitreum*) and its European congener, the pikeperch (*Sander lucioperca*). Although the largemouth bass is native to North America only, it has been introduced in many parts of Europe, and most of the European waterbodies in our database contained largemouth bass if they had any piscivores at all.

For assessing competitive pressure, we considered only congeneric competitors, and used the presence or absence of other lepomids in the waterbody as an indicator of this pressure. The bluegill (*Lepomis macrochirus*) in particular is considered the major competitor against the pumpkinseed in North America (e.g., Werner and Hall 1976; Keast 1978; Mittelbach 1988), and its absence has been shown to affect morphological diversification in pumpkinseed (Robinson et al. 2000). Aside from the pumpkinseed, sunfishes of the genus *Lepomis* are rare in European waterbodies and not present in any of our study sites, so life-history traits in the presence or absence of these competitors was only used to disentangle life-history traits associated with competitors and predators within North America.

### Data analysis

To determine whether there were significant differences in life-history traits between native North American and introduced European pumpkinseed populations, we first compared mean age and length at maturity and GSI, as well as juvenile growth rate (mean length at age 2) in populations from the two continents with independent *t* tests. To test the hypothesis that pumpkinseed life-history traits will be related to the rate of juvenile growth, the thermal environment, and the size of the waterbody, and to test the prediction that introduced populations would show more opportunistic life-history traits than native North American populations when these variables were accounted for, we used an information theoretic approach (Burnham and Anderson 2002) to compare linear regression models using all combinations of four variables (length at age 2, air temperature degree-days, surface area of the waterbody, and continental origin) to predict each of the three life-history traits. In the case of surface area, we used log_e_-transformed data in the models to correct for non-normality. To evaluate the models, we calculated Akaike’s information criterion corrected for small sample bias (AICc), The best model for each life-history trait was considered to be that with the lowest AICc. Those with an AICc difference (∆_*i*_) < 2 from the best model were considered to have strong support, whereas those with 2 < ∆_*I*_ < 4 were considered to have moderate support (Burnham and Anderson 2002). To guard against multi-colinearity, we tested for tolerance between the two continuous variables. In all cases, tolerance was >0.4, well above the 0.1 acceptability criterion (Quinn and Keough 2002).

To test the hypothesis that the presence/absence of piscine predators will affect life-history traits on both continents, we conducted two-way analyses of variance of the three pumpkinseed life-history traits using continent and present/absence of obligate piscivores as main factors, followed by a Tukey HSD test to compare traits in North American and European populations with piscivores, to test the prediction that intercontinental differences in pumpkinseed life-history traits would not be manifested in the presence of piscivores. This was followed by discriminant function analysis in order to determine whether groups of populations classified by continent and the presence and absence of piscivores would be segregated from one another by a combination of life-history traits plus juvenile growth rate.

While the influence of major competitors could not be assessed in the same way as the influence of predators due to the absence of lepomid competitors in our European waterbodies, we attempted to compare their influence with that of predators by classifying populations into five groups according to continent, presence/absence of piscine predators and presence/absence of lepomid competitors. One-way analysis of variance was then used to assess differences in the three life-history traits among the five groups, followed by Tukey HSD tests to determine which groups were different. A discriminant function analysis was then conducted on these groups using the same combination of growth and life-history traits that was used in the analysis of predator presence/absence. The level of statistical significance for all tests was set at *P* < 0.05.

## Results

European pumpkinseed populations matured earlier and at a smaller size, and had higher GSIs than North American populations, despite showing no significant differences in juvenile growth rate or air temperature degree-days (Table 2). North American waterbodies containing pumpkinseed were significantly larger in surface area than those in Europe, where a number of the study populations inhabit artificial ponds.

**Table 2:** Comparison of growth and life-history traits and environmental attributes (mean ± SE) of native North American and introduced European waterbodies containing pumpkinseed [mean total length (*TL*) at age 2 compares populations with a mean age of maturity >2 years]

Characteristic	North America	Europe	*P*
Growth and life-history traits			
Mean age at maturity (year)	2.98 (0.15)	2.21 (0.10)	<0.001
Mean TL at maturity (mm)	99.4 (2.8)	77.0 (1.4)	<0.001
GSI (%)	5.8 (0.33)	8.7 (0.35)	<0.001
Mean TL at age 2 (year)	73.3 (2.35)	76.6 (2.72)	0.36
Environmental attributes			
Surface area (m^2^; log_e_-transformed)	4.94 (0.43)	2.0 (0.66)	<0.001
Degree-days	1,284 (75)	1,303 (102)	0.88

Significance of differences was assessed with independent *t* tests

The strongest models for predicting mean age and length at maturity included continental origin, mean length at age 2, and surface area of the waterbody as independent variables (Table 3; Appendix 2). This combination of variables accounted for 57 % of the variation in mean age at maturity and 39 % of the variation in mean length at maturity. For the prediction of age at maturity, the sign of all coefficients were uniform, with age at maturity declining as length at age 2 increases and as surface area decreases. Age at maturity also increased with air temperature degree-days, but this variable was unstable in the model (SE > coefficient; low *w*_*i*_) and it was not found with strong support in either of the two models. For the prediction of mean length at maturity, the sign of the coefficients were also uniform with a single exception (mean length at age in the model with continental origin). Length at maturity increased with both surface area and temperature.

**Table 3:** Model selection results from an analysis of the effects of continental origin (continent), mean length at age 2 (*L2*), air temperature degree-days (*temp*), and log_e_-transformed surface area of the waterbody (*area*) on three life-history traits of pumpkinseed populations

Trait	Independent variable(s)	*K*	AICc	Relative likelihood	∆_*i*_	*w*_*i*_	$R^{ 2}_{\text{adj}}$
Age at maturity	Continent, L2, area	5	140.78	1.0	0.00	0.48	0.57
	Continent, L2	4	141.87	0.58	1.11	0.27	0.56
	Continent, L2, area, temp	6	143.16	0.30	2.38	0.14	0.56
	Continent, L2, temp	5	143.79	0.22	3.01	0.11	0.55
Length at maturity	Continent, L2, area	5	558.75	1.0	0.00	0.36	0.39
	Continent, L2	4	558.92	0.92	0.17	0.34	0.38
	Continent, L2, temp	5	560.96	0.33	2.21	0.12	0.33
	Continent, L2, area, temp	6	561.09	0.31	2.34	0.11	0.31
GSI	Continent, L2	4	279.62	1.0	0.00	0.42	0.40
	Continent	3	281.85	0.33	2.23	0.14	0.40
	Continent, L2, temp	5	281.85	0.33	2.23	0.14	0.39
	Continent, L2, area	5	281.89	0.32	2.27	0.13	0.39
	Continent, temp	4	283.57	0.14	3.94	0.06	0.39

Candidate models are ranked by the Akaike information criterion corrected for small sample size (AICc), with the differences between models shown as ∆_*i*_. All combinations of the four variables were assessed; the models displayed are those classified as strong (∆_*i*_ ≤ 2) and moderate (4 ≤ ∆_*i*_ ≤ 2). Also shown for each model are the number of parameters (*K*), Akaike weight (*w*_*i*_), relative likelihood, and proportion of variation explained by the model, adjusted for the number of variables ($R^{ 2}_{\text{adj}}$)

The only strong model for predicting GSI had continental origin and mean length at age 2 as independent variables (Table 3). The signs of coefficients were consistent across models with the exception of air temperature degree-days, which was included in two of the models with only moderate support and was unstable in both of these models. GSI increased with length at age 2 and surface area.

Across the three life-history traits, all models with strong and moderate support included continental origin. These models demonstrate that European pumpkinseed populations mature earlier and at a smaller size, and have a higher GSI than North American populations, even when juvenile growth, size of the waterbody, and temperature are accounted for.

**Fig. 1:**
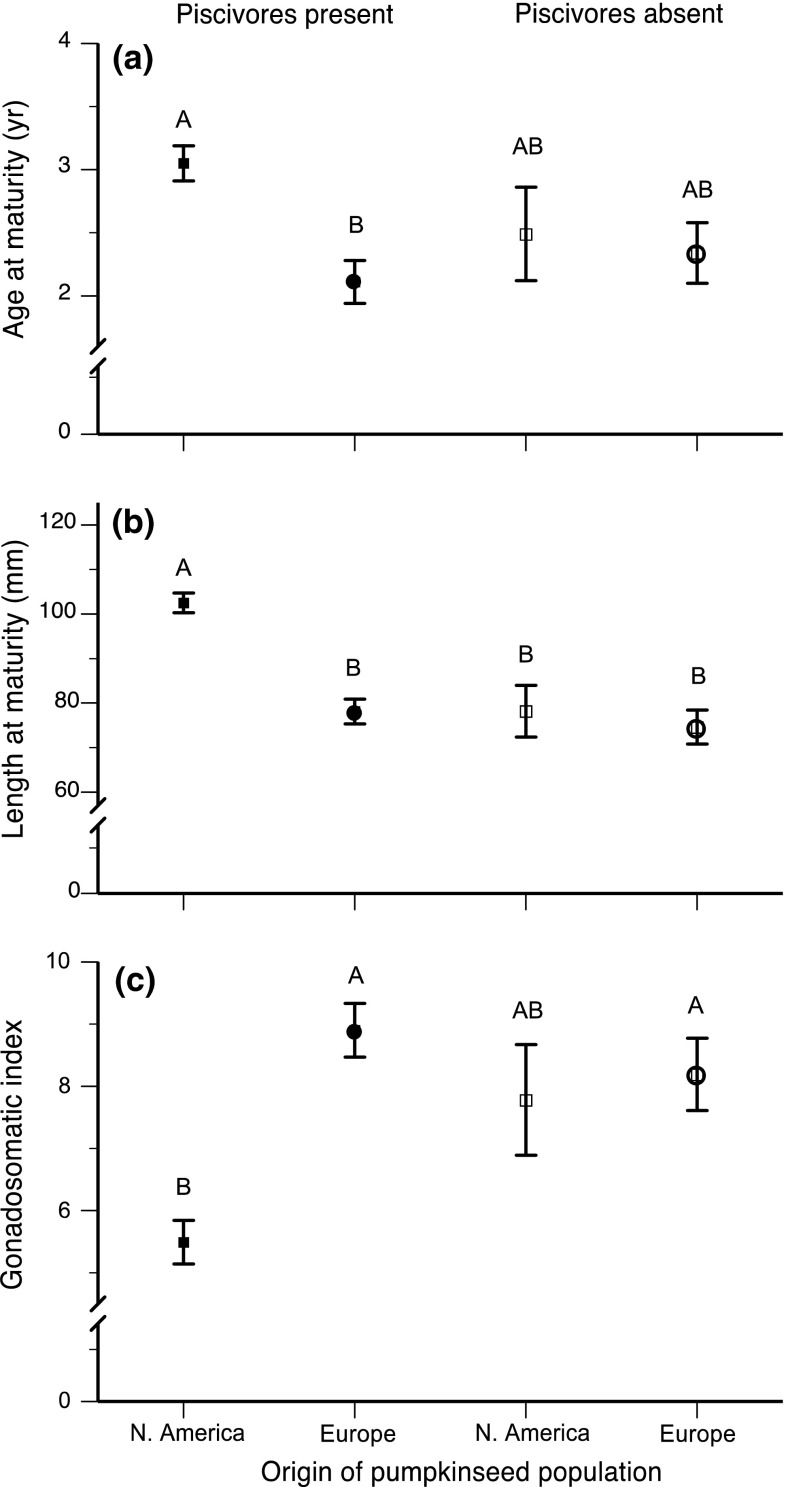
Comparison of three life-history traits in North American (*squares*) and European (*circles*) pumpkinseed (*Lepomis gibbosus*) populations (females only) in the presence (*solid symbols*) and absence (*open symbols*) of piscivorous fishes (*left* and* right of the panels*, respectively): **a** age at maturity; **b** length at maturity; **c** gonadosomatic index. *Data points* shown are mean ± SE; the number of populations studied were: **a** (40, 28, 6, 15); **b** (40, 25, 6, 15); **c** (39, 26, 6, 14) from* left* to* right* in each panel. Means with a different* letter* are significantly different (Tukey HSD test, *P* < 0.05)

Two-way analysis of variance models examining the effects of continent and the presence/absence of predators were highly significant for all three life-history traits in the overall models, and while continental origin was a significant factor in all of the models (*P* < 0.04 in all cases), there were significant continent by predator interactions for length at maturity and GSI. A comparison of populations by continent shows that when predators were absent, there were no significant differences in age at maturity, size at maturity, or GSI between North American and European pumpkinseed populations. In contrast, in the presence of predators, North American populations matured significantly later, at a greater TL, and with a lower GSI than European populations (Fig. 1).

**Fig. 2:**
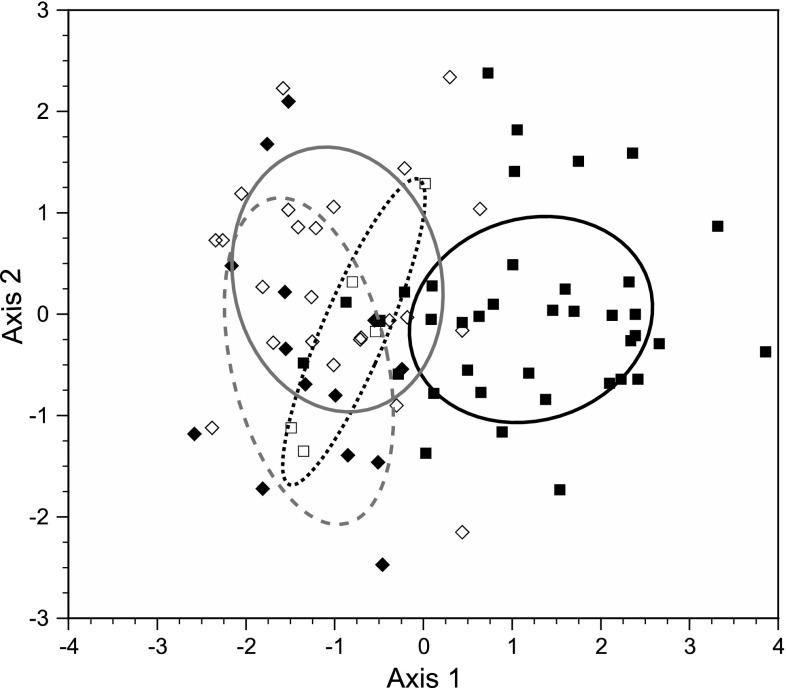
Distribution of pumpkinseed scores from the discriminant function analysis of life-history and juvenile growth traits of pumpkinseed populations, segregated by continent and piscivore presence/absence, with 50 % ellipoids around the centroid of each group, plotted on the first two canonical axes. Key to symbols and ellipsoids: native North American pumpkinseed populations in the presence of piscivores (*solid squares* and *solid black* line, *n* = 38) and in the absence of piscivores (*open squares* and *dotted line*, *n* = 6); introduced European populations in the presence of piscivores (*solid diamonds*, *solid gray line*, *n* = 24), and in the absence of piscivores (*open diamonds*, *dashed line*, *n* = 14). Refer to Table 4 for axis descriptions

In discriminant function analysis, the first two axes explained over 99 % of the variation among populations. The first axis (94 % of total variation) showed a strong positive correlation with mean length at maturity and a moderate negative correlation with mean GSI of pumpkinseeds, and the second axis (5 % of total variation) showed moderate positive correlations with mean length at age 2 and mean GSI (Table 4). A scatterplot of populations revealed strong segregation along axis 1 of North American populations in the presence of obligate piscivores from those lacking piscivores, as well as from European populations in the presence or absence of piscivores (Fig. 2). Mahalanobis distances generated from the axis scores of all sites differentiated North American populations in the presence of piscivores from all other groups (*P* < 0.0016 in all cases), whereas there was no significant differentiation among the other groups (*P* > 0.14 in all cases).

**Table 4:** Standardized canonical coefficients and correlations between life-history and growth means of pumpkinseed populations and the first two canonical axes of the discriminant function in an analysis by continent and piscivore presence/absence

Parameter	Axis 1		Axis 2	
	Coefficient	Correlation	Coefficient	Correlation
Mean age at maturity	0.911	0.42	0.916	−0.45
Mean TL at maturity	0.255	0.82	−0.247	−0.04
Mean GSI	−0.394	−0.62	0.690	0.65
Mean TL at age 2	0.950	0.18	1.341	0.71
Eigenvalue	1.345		0.072	
Cum. proportion	0.944		0.995

**Fig. 3:**
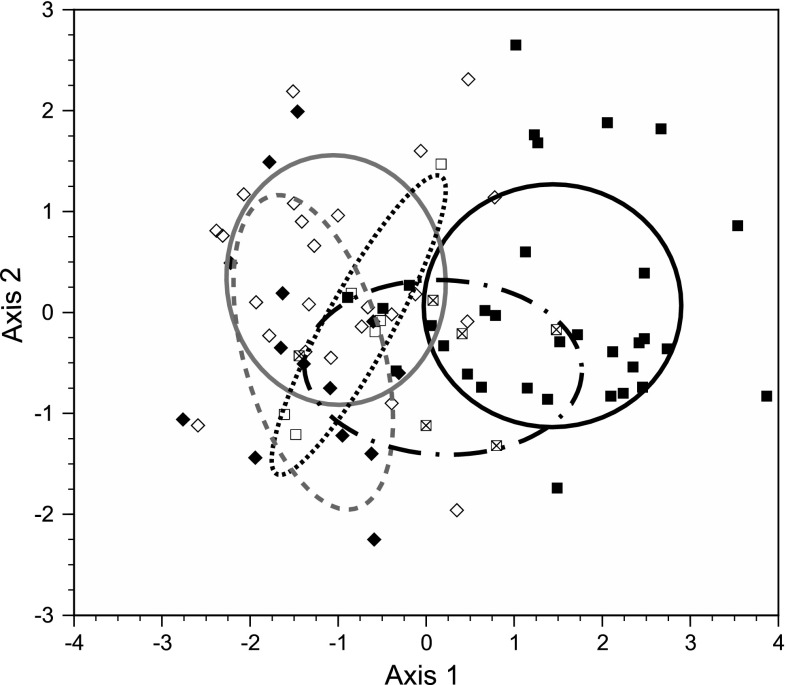
Distribution of pumpkinseed scores from discriminant function analysis of life-history and juvenile growth traits of pumpkinseed populations, segregated by continent, piscivore presence/absence, and the presence/absence of other lepomid species. Population data points are plotted on the first two canonical axes with 50 % ellipsoids around the centroid of each group. Key to symbols and ellipsoids: native North American pumpkinseed populations in the presence of piscivores and other lepomids (*solid squares* and *solid black line*, *n* = 32), in the presence of piscivores with other lepomids absent (*squares with cross inside* and *dotted-dashed line*, *n* = 6), and in the absence of piscivores and other lepomids (*open squares* and *dotted line*, *n* = 6); introduced European populations in the presence of piscivores (*solid diamonds*, *solid gray line*, *n* = 24), and in the absence of piscivores (*open diamonds*, *dashed line*, *n* = 14). Note that other lepomid species were not present in the European waterbodies, and none of the native waterbodies contained other lepomids but no piscivores. Refer to Table 5 for axis descriptions

When North American pumpkinseed populations were segregated into three groups (obligate piscivores and other lepomids present, obligate piscivores present and other lepomids absent, and both piscine piscivores and other lepomids absent), and life-history traits were then compared with European pumpkinseed populations with or without piscine predators using analysis of variance, only North American populations living sympatrically with both piscivores and other lepomids were significantly different in mean age at maturity than European populations with piscivores, and significantly different in mean length at maturity and GSI than European populations with or without piscivores (Tukey HSD, *P* < 0.05 in all cases). In discriminant function analysis, the first axis (94 % of total variation) showed a moderate positive correlation with mean length at maturity and a moderate negative correlation with mean GSI, and the second axis (5 % of total variation) showed moderate positive correlations with mean length at age 2 and mean GSI (Table 5). A scatterplot of populations revealed strong segregation along axis 1 of North American populations in the presence of obligate piscivores and other lepomids from those containing neither piscivores nor other lepomids, as well as from European populations in the presence or absence of piscivores. North American populations in waterbodies containing piscivores but no other lepomids showed a moderate to strong life-history overlap with all other groups (Fig. 3).

**Table 5:** Standardized canonical coefficients and correlations between life-history and growth means of pumpkinseed populations and the first two canonical axes of the discriminant function in an analysis by continent, piscivore presence/absence and presence/absence of other lepomids

Parameter	Axis 1		Axis 2	
	Coefficient	Correlation	Coefficient	Correlation
Mean age at maturity	1.021	0.37	0.902	−0.57
Mean TL at maturity	0.209	0.78	−0.373	−0.18
Mean GSI	−0.394	−0.58	0.578	0.62
Mean TL at age 2	1.098	0.21	1.386	0.79
Eigenvalue	1.567		0.092	
Cum. proportion	0.936		0.991

## Discussion

Our results clearly demonstrate that life-history traits of pumpkinseed introduced into European waterbodies differ significantly from those of native North American populations. European pumpkinseed populations displayed earlier maturity, maturity at a smaller size, and greater reproductive investment than those of North America, and thus show a more opportunistic suite of life-history traits than native populations. The differences are evident even when growth prior to maturity, size of the waterbody, and thermal regime are taken into account.

The more opportunistic life-history traits displayed by European non-native pumpkinseed populations are consistent with those predicted for low-density fish populations experiencing high resource availability (Weeks 1993), and have been exhibited by other introduced fishes during their range expansion phase (Museth et al. 2002; Bøhn et al. 2004; Gutowsky and Fox 2012). However, the introduction of pumpkinseed in Europe has occurred over at least a century (Vivier 1951; Lever 1996), and, while some populations continue to grow and expand rapidly, many others are at moderate to high densities but are not expanding their range (Copp and Fox 2007; Vila-Gispert et al. 2007; Van Kleef et al. 2008). Given the high variability in length of establishment of European pumpkinseed populations, and the absence of significant differences in juvenile growth rate between European and North American populations, it seems highly unlikely that the present-day intercontinental differences in life-history traits are the result of a plastic response to lower population density or higher resource availability in European waterbodies.

A more plausible explanation for the life-history differences between North American and European populations involves natural selection for opportunistic life-history traits operating on small European founder populations. Species introductions frequently result in large reductions in allelic richness and heterozygosity relative to that of source populations (reviewed in Dlugosh and Parker 2008). Under these circumstances, the initial low pumpkinseed densities, combined with the absence of major competitors and reduced predation risk (discussed below), could have resulted in strong selection pressure on pumpkinseed life-history traits. Although there have been shifts toward less opportunistic life-history traits in some species of introduced fishes as they became established over time (Bøhn et al. 2004; Feiner et al. 2012; Gutowsky and Fox 2012), any such shifts in European pumpkinseed have not made their life histories comparable to those of native populations. The combination of reduced predation risk relative to native populations and the absence of native congeneric competitors in European waters may have been strong enough to maintain the selection for opportunistic life-history traits, even though higher densities, as the pumpkinseed became more established, would have favored a regression of life-history traits back to that of native populations.

Our hypothesis that pumpkinseed life-history traits would be related to the rate of juvenile growth, the thermal environment, and the size of the waterbody was mostly supported, in that all three independent variables were included in candidate models with strong and moderate support. The strongest candidate models for predicting these life-history traits all contained mean length at age 2, the juvenile growth indicator, as one of the terms. Surface area of the waterbody was included in the best candidate models for predicting age and length at maturity but not GSI, and air temperature degree-days was only part of candidate models with moderate support (Table 3). The negative relationship between juvenile growth and age at maturity had been previously demonstrated in pumpkinseed populations on both continents, albeit on a more limited geographic scale in North America (native populations in Ontario, Canada: Fox 1994; non-native UK populations: Villeneuve et al. 2005; UK and continental Europe: Cucherousset et al. 2009). The idea of earlier maturity of faster-growing individuals has also been supported by life-history model predictions (e.g., Stearns and Koella 1986) as well as by empirical studies of other fishes (e.g., Alm 1959; Pitt 1975; Hutchings 1993). In pumpkinseed, this relationship has been proposed as a biological predictor of invasiveness in European populations (Copp and Fox 2007), and subsequently acquired data points for other European pumpkinseed populations have so far fit this model (Valente 2008; Cucherousset et al. 2009; Fobert et al. 2013).

The negative relationship between temperature and age at maturity has been previously documented in many ectotherms (reviewed in Atkinson 1994) including fishes (reviewed in Gillooly et al. 2002). In the case of the pumpkinseed, the influence of temperature on age at maturity had been previously inferred by comparing populations from thermal extremes (Fox and Crivelli 2001; Dembski et al. 2006; Fox et al. 2007) or by using latitude as a proxy for thermal regime (Cucherousset et al. 2009). The influence of both temperature and juvenile growth rate on age at maturity are likely to be interrelated in a warmwater species like the pumpkinseed, as higher temperatures typically reduce development time and increase the rate of juvenile growth (reviewed by Atkinson 1994). Air temperature degree-days was negatively correlated with mean age at maturity in our study sites as would be expected (*r* = −0.55, *P* < 0.001), but the relationship was weaker than that exhibited by other variables tested in our models to predict age at maturity. The relationship may have been stronger had water temperature data been available instead of air temperature for the study sites.

The reduced ability of the best candidate models to account for length at maturity relative to the best models accounting for age at maturity can be partly explained by the effects of the independent variables on age at maturity. Size at maturity depends on growth rate and age at maturity, so maturity at an earlier age may offset the effect of faster juvenile growth or warmer temperatures on the size at which maturity occurs. According to the life-history model of Stearns and Koella (1986), the reaction norm between age and size at maturity depends upon the relationship between growth and mortality rates in the juvenile and adult life stages. In this model, age at maturity is more responsive to changes in somatic growth rates than size at maturity when either adult mortality or both juvenile and adult mortality increase with a decrease in growth rate (trajectories 3 and 4 in Stearns and Koella 1986, fig. 6). Although no studies are available to test the effect of growth rate on mortality rates in pumpkinseed, adult mortality rates have been found to increase in smaller-bodied native populations (Bertschy and Fox 1999), and overwinter mortality in smaller-bodied YOY was shown to be higher than in larger-bodied YOY (Bernard and Fox 1997). Empirical support for the absence of a juvenile growth effect on GSI has also been shown in a comparative life-history study of 27 native pumpkinseed populations, where mean length at age 2 was significantly correlated with mean age at maturity, but not with mean GSI in these populations (Fox 1994).

Given the differences in life-history traits in the presence or absence of piscivores in the native range, but the lack of such differences in the non-native range, the more opportunistic life-history traits of European pumpkinseed could be explained if some level of release from predation was involved (reviewed by Colautti et al. 2004). Pumpkinseed in Europe are eaten by obligate piscivores that are also present in North America such as the northern pike (Guti et al. 1991; Copp et al. 2010) and the introduced largemouth bass (Godinho et al. 1997). Pumpkinseed are also eaten by facultative piscivores that are congeneric equivalents to North American species, such as the European eel (*Anguilla anguilla*) and the Eurasian perch (*Perca fluviatilis*) (Copp et al. 2010). However, the fish communities of European waterbodies are frequently dominated by cyprinids (Reyjol et al. 2007), and being more fusiform in shape than lepomids and lacking their sharp dorsal and anal fin spines, cyprinids are easier to handle and consume than lepomids like the pumpkinseed (Hoyle and Keast 1987). Furthermore, centrarchids such as the pumpkinseed are a much more abundant prey to obligate piscivores of their native range than in Europe, in particular in more productive systems, which characteristically have a lower proportion of obligate piscivores (Persson 2008). The high vulnerability of cyprinids to bass predation is indicated in a study of lake populations in Ontario, Canada, where lakes containing largemouth and smallmouth bass showed diminished richness of cyprinid species relative to lakes not containing these species (Jackson 2002). There is also evidence from several European studies that the largemouth bass consumes far less pumpkinseed than other fish and invertebrate prey types, despite the prevalence of the pumpkinseed in these waterbodies (e.g., Rodríguez-Jiménez 1989; García-Berthou 2002; Lorenzoni et al. 2002; Marinelli et al. 2007).

The Enemy Release Hypothesis relates not only to the release of non-native species from native predators but also from competitors (Keane and Crawley 2002; Colautti et al. 2004). While our study provides strong support for the Enemy Release Hypothesis, it does not fully disentangle the potential effects of release from predators from those of competitors. Life-history traits of native pumpkinseed populations with both obligate piscivores and other lepomids were clearly separated in multivariate life-history space from Europe populations, whereas the traits of native populations with piscivores, but no other lepomids, showed a greater overlap with those of European populations (Fig. 3). Unfortunately, there were only six native populations in the latter category, and we were unable to collect life-history data on any native populations with obligate piscivores absent and other lepomids present, so we could not contrast the absence of native competitors from that of native predators. However, the results of these comparisons do suggest that both the reduction in predation rates and the absence of lepomid competitors in Europe play a role in the manifestation of more opportunistic life-history traits in European pumpkinseed, albeit by different mechanisms.

Predators have been attributed as a major influence on the life-history traits of a number of aquatic taxa, including daphnids (Spitze 1991; Borcic et al. 1998), snails (Crowl and Covich 1990), amphipods (Wellborn 1994), and fish (Reznick and Endler 1982; Belk and Hales 1993; Rennie et al. 2010). One mechanism that could explain how reduced predation on European pumpkinseed leads to more opportunistic life-history traits on that continent is a shift in energy allocation under reduced juvenile predation risk. Several researchers have hypothesized that in response to heavy predation by gape-limited predators on small individuals, prey species will delay the expenditure of energy on reproduction until they grow large enough to pass through their window of vulnerability (reviewed in Day et al. 2002). The “window of vulnerability” hypothesis would predict that relaxed predation rate would change the balance of fitness trade-offs away from delaying maturity in favor of somatic growth and towards increased energy expenditures for reproduction early in life, which is what is observed in European pumpkinseed populations.

The absence of major competitors might be expected to affect pumpkinseed life-history traits through an increase in energy availability. The bluegill in particular co-occurs with the pumpkinseed over much of its native range (see Scott and Crossman 1973), and the two species have been shown to eat many of the same prey types and to compete extensively for food resources (Werner and Hall 1976; Keast 1978; Mittelbach 1988). Osenberg et al. (1992) developed a two-stage life-history model using these species, and showed with empirical data how the release of pumpkinseed from competition with bluegill in the juvenile stage can lead to fast growth at this stage, but reduced resource availability, increased competition for the prey juveniles normally consume, and reduced growth at the adult stage. In a comparison of life-history traits of Ontario pumpkinseed populations living in sympatry or allopatry with bluegill, Fox (1994) showed that allopatric populations matured earlier at a smaller size and had increased energy allocation relative to sympatric populations. This pattern is consistent with that observed in European populations in which bluegill and other lepomid species are absent; however, if the mechanism is release from juvenile competition, one would expect European juveniles to be larger than North American juveniles, at least until the North American juveniles get large enough to feed on adult resources such as gastropods (see Osenberg et al. 1992). As in the Ontario study, North American–European differences in mean length at age 2 are in the direction consistent with a release-from-competition explanation, but the difference in length is not significant. Thus, our study provides strong support for the hypothesis that the more opportunistic life-history traits of European pumpkinseed can be attributed to some form of enemy release, but whether this is primarily due to reduced predation or release from native competitors cannot be determined without controlled experiments.

## Electronic supplementary material

Below is the link to the electronic supplementary material.

Supplementary material 1 (DOC 163 kb)

Supplementary material 2 (DOC 96 kb)
