# Supplementary material for: Old world versus new world: life-history alterations in a successful invader introduced across Europe
Source: Oecologia. 2013 Sep 25;174(2):435–46. doi: 10.1007/s00442-013-2776-7 (PMC3897869; doi:10.1007/s00442-013-2776-7)
Supplement: Supplementary file 1 — Supplementary material 1 (DOC 163 kb) [file 442_2013_2776_MOESM1_ESM.doc]

**Appendix 1** Location and surface area of native North American (NA) and non-native European (EU) water bodies containing pumpkinseed used in this study.

| Loc^a^ | Latitude longitude | Water body | Area (ha) | Obligate piscivores^b^ | Source |
| --- | --- | --- | --- | --- | --- |
| North American native populations | | | | |  |
| ON | 44˚34'N 78˚50'W | Balsam Lake | 4 665 | LMB, MU, WA | Fox (1994) |
| ON | 44˚32'N 78˚25'W | Buckhorn Lake | 3 191 | LMB, MU, WA | Fox (1994) |
| ON | 44˚07'N 78˚53'W | Scugog Lake | 6 734 | LMB, MU, WA | Fox (1994) |
| ON | 44˚10'N 78˚10'W | Rice Lake | 10 018 | LMB, MU, WA | Fox (1994) |
| ON | 44˚49'N 76˚44'W | Warrens Lake | 34 | LMB, MU, WA | Fox (1994); Bertschy and Fox (1999) |
| ON | 44˚48'N 76˚41'W | Little Round Lake | 7.5 | - | Fox (1994); Bertschy and Fox (1999) |
| ON | 44˚32'N 76˚24'W | Lower Poole Pond | 2.7 | - | Fox (1994); Fox and Crivelli (2001) |
| ON | 44˚27'N 77˚57'W | Beloporine Lake | 7.2 | - | Fox (1994) |
| ON | 44˚47'N 76˚43'W | Black Lake | 39.6 | LMB, NP, WA | Fox (1994) |
| ON | 44˚32'N 76˚22'W | Lower Dowsley Pond | 2.6 | - | Fox and Keast (1991); Fox (1994) |
| ON | 44˚31'N 77˚49'W | Upper Poole Pond | 0.7 | - | Fox and Keast (1991); Fox (1994) |
| ON | 45˚34'N 80˚23'W | Shawanaga River | - | LMB, NP, WA | Fox (1994) |
| ON | 44˚21'N 76˚12'W | Lake Opinicon | 787 | LMB, NP | Fox (1994) |
| ON | 44˚59'N 77˚58'W | Vance Lake | 8 | LMB, NP | Fox (1994); Bertschy and Fox (1999) |
| ON | 45˚19'N 75˚42'W | Rideau River | - | LMB, NP, WA | Unpublished data (Ferguson and Fox) |
| ON | 44˚43'N 76˚56'W | Arden Lake | 13.8 | LMB, NP | Fox (1994) |
| ON | 44˚31'N 77˚49'W | Bass Lake | 290 | LMB, NP, WA | Fox (1994) |
| ON | 44˚35'N 76˚42'W | Duncan Lake | 44.6 | LMB, WA | Fox (1994) |
| ON | 44˚48'N 76˚15'W | Long Lake | 85 | LMB | Fox (1994) |
| ON | 44˚23'N 76˚28'W | Loughborough Lake | 1 804 | LMB, NP | Fox (1994) |
| ON | 44˚36'N 76˚08'W | Lower Beverly Lake | 766 | LMB | Deacon and Keast (1987) |
| ON | 44˚45'N 76˚44'W | McLeans Lake | 8.1 | LMB, NP | Fox (1994) |
| ON | 44˚47'N 76˚08'W | Otter Lake | 572 | LMB | Fox (1994) |
| ON | 44˚51'N 76˚43'W | Pennick Lake | 20.3 | - | Fox (1994) |
| ON | 44˚30'N 77˚52'W | Round Lake | 568 | LMB | Unpublished data, M. Fox |
| ON | 44˚33'N 76˚15'W | Smiths B Lake | 7.1 | LMB | Fox (1994) |
| ON | 44˚38'N 76˚05'W | Upper Beverly Lake | 551 | LMB | Deacon and Keast (1987) |
| ON | 44˚30'N 76˚25'W | Upper Rock Lake | 72.8 | LMB | Fox (1994) |
| ON | 44˚29'N 77˚44'W | Crowe Lake | 1 037 | LMB, WA | Fox (1994) |
| ON | 44˚40'N 77˚53'W | East Twin Lake | 6.8 | LMB, NP | Unpublished data, M. Fox |
| ON | 44˚31'N 77˚49'W | Belmont Lake | 770 | LMB, MU, WA | Unpublished data, M. Fox |
| ON | 44˚15'N 78˚10'W | Indian River | - | MU, SMB | Brinsmead (2000) |
| NY | 43˚11'N 75˚59'W | Oneida Lake | 20 668 | LMB, WA, NP | This study |
| NY | 42˚41'N 76˚41'W | Cayuga Lake | 17 319 | LMB, WA, NP | This study |
| PA | 41˚21'N 76˚03'W | Harveys Lake | 266 | LMB, WA | This study |
| PA | 40˚47'N 76˚07'W | Locust Lake | 21 | LMB, WA | This study |
| NJ | 39˚31'N 75˚08'W | Parvin Lake | 38 | LMB | This study |
| NJ | 39˚23'N 75˚01'W | Union Lake | 364 | LMB | This study |
| VA | 36˚54'N 77˚01'W | Lake Airfield | 43 | LMB | Fox et al. (2007) |
| VA | 36˚51'N 76˚02'W | Lake Smith | 78 | LMB | Fox et al. (2007) |
| VA | 36˚54'N 76˚11'W | Lake Whitehurst | 185 | LMB | Fox et al. (2007) |
| VA | 38˚35'N 77˚15'W | Powells Creek | - | LMB | Fox et al. (2007) |
| VA | 38˚36'N 77˚15'W | Neabsco Creek | - | LMB | Fox et al. (2007) |
| NC | 36˚12'N 76˚28'W | Perquimans River | - | LMB | Fox et al. (2007) |
| NC | 36˚04'N 76˚26'W | Yeopim River | - | LMB | Fox et al. (2007) |
| NC | 35˚47'N 76˚29'W | Lake Phelps | 6 721 | LMB | Fox et al. (2007) |
| European non-native populations | | | | |  |
| GB | 51˚01'N 00˚04'E | Boringwheel Lake | 2 | NP | Villeneuve et al. (2005) |
| GB | 51˚01'N 00˚04'E | Batts Bridge Stream | - | NP | Villeneuve et al. (2005) |
| GB | 50˚59'N 00˚04'E | Powdermill Upper Pond | 0.1 | NP | Villeneuve et al. (2005) |
| GB | 50˚59'N 00˚04'E | Powdermill Side Pond | 0.1 | NP | Villeneuve et al. (2005) |
| GB | 50˚59'N 00˚04'E | Powdermill Lower Pond | 2.8 | NP | Villeneuve et al. (2005) |
| GB | 51˚01'N 00˚00'E | Tanyards Pond 2 | 0.4 | - | Villeneuve et al. (2005) |
| GB | 51˚01'N 00˚00'E | Tanyards Pond 3 | 0.4 | - | Villeneuve et al. (2005) |
| GB | 51˚01'N 00˚00'E | Tanyards Pond 4 | 0.3 | - | Villeneuve et al. (2005) |
| GB | 51˚01'N 00˚00'E | Tanyards Pond 5 | 0.5 | - | Villeneuve et al. (2005) |
| GB | 51˚01'N 00˚00'E | Tanyards Pond 6 | 0.4 | - | Villeneuve et al. (2005) |
| GB | 51˚01'N 00˚00'E | Tanyards Pond 7 | 0.5 | NP | Villeneuve et al. (2005) |
| GB | 51˚07'N 00˚09'W | Milton Mount Pond | 1.4 | NP | Villeneuve et al. (2005) |
| GB | 51˚05'N 00˚13'W | Cottesmore School Pond | 1.5 | NP | Copp et al. (2002) |
| GB | 51˚05'N 00˚13'W | Cottesmore Middle Pond | 0.5 | - | Villeneuve et al. (2005) |
| GB | 51˚05'N 00˚13'W | Cottesmore Lower Pond | 0.5 | - | Villeneuve et al. (2005) |
| GB | 51˚06'N 00˚13'W | Island Pond | 1.0 | NP | Villeneuve et al. (2005) |
| GB | 51˚06'N 00˚13'W | Douster Pond | 2.1 | NP | Villeneuve et al. (2005) |
| GB | 51˚07'N 02˚58'W | Dunwear Pond | 2.2 | NP | Villeneuve et al. (2005) |
| GB | 50˚39'N 01˚11˚W | Airport Pond | 0.7 | - | Villeneuve et al. (2005) |
| SP | 37˚55'N 05˚00'W | Guadiato River | - | LMB | Gutiérrez-Estrada et al. (2000 and unpubl.) |
| SP | 42˚08’N 02˚45’E | Lake Banyoles | 112 | LMB | Fox et al. (2007) |
| SP | 41˚58’N 02˚30’E | Susqueda Reservoir | 466 | LMB | Fox et al. (2007) |
| SP | 42˚21’N 02˚48’E | Boadella Reservoir | 364 | LMB, NP, PP | Fox et al. (2007) |
| SP | 41˚18’N 00˚21’E | Riba-roja Reservoir | 2 152 | LMB | Fox et al. (2007) |
| SP | 42˚12'N 03˚06’E | Fluvia River | - |  | Fox et al. (2007) |
| SP | 42˚15’N 03˚06’E | Muga River | - | NP | Fox et al. (2007) |
| SP | 42˚01’N 03˚12’E | Ter River | - |  | Fox et al. (2007) |
| FR | 43˚30'N 04˚40'E | Fumemorte Canal | - | - | Fox and Crivelli (2001) |
| FR | 43˚28'N 04˚54'E | Sollac Marsh | - | LMB | Fox and Crivelli (2001) |
| FR | 43˚32'N 04˚45'E | Ligagneau Canal | - | - | Fox and Crivelli (2001) |
| FR | 43˚21'N 04˚29'E | Rhone River | - | NP, PP | Unpublished data, M. Fox and A. Crivelli |
| H | 47˚11'N 19˚13'E | Dabas Pond | - | n/a | Tandon (1977) |
| GR | 41˚11'N 23˚08'E | Kerkini Reservoir | 5 940 | PP | Neophitou and Giapis (1994) |
| RO | 45˚18'N 28˚09'E | Danube Delta | - | PP | Papadopol and Ignat (1967) |
| RO | 44˚36'N 27˚10'E | Fundata Lake | 969 | - | Constantinescu (1981) |
| SK | 48˚06'N 17˚29'E | Danube R.side channel | - | NP, PP | This study |
| NL | 51˚23'N 05˚30'E | Meeuwven Pond | 3 | - | Cucherousset et al. (2009) |
| B | 50˚58'N 05˚05'E | Webbekomsbroek Pond | 2 | NP | Cucherousset et al. (2009) |
| B | 50˚57'N 05˚18'E | Slangebeek Stream | - | - | Cucherousset et al. (2009) |
| FR | 47˚22'N 02˚19'W | Brière Marsh | 9 000 | LMB, NP, PP | Cucherousset et al. (2009) |
| FR | 47˚05'N 01˚39'W | Grand-Lieu Lake | 4 000 | LMB, NP, PP | Cucherousset et al. (2009) |
| SP | 38˚17'N 01˚41'W | Segura River | - | LMB, PP | This study |
| SP | 38˚10'N 01˚21'W | Ojos Reservoir | - | LMB, PP | This study |
| SP | 41˚23’N 00˚55’E | Flix Reservoir | 320 | LMB, PP | This study |

^a^ Location of North American populations: NC = North Carolina, NJ = New Jersey, NY = New York, PA = Pennsylavania, ON = Ontario, VA = Virginia. European populations: B = Belgium, FR = France, GB = Great Britain, GR = Greece, H = Hungary, NL = Netherlands, RO = Romania, SK = Slovakia, SP = Spain

^b^Obligate piscivores: LMB = largemouth bass (*Micropterus salmoides*), MU = muskellunge (*Esox masquinongy*), NP = northern pike (*Esox lucius*), PP = pikeperch (*Sander lucioperca*), WA = walleye (*Sander vitreum*)
