# Supplementary material for: Old world versus new world: life-history alterations in a successful invader introduced across Europe
Source: Oecologia. 2013 Sep 25;174(2):435–46. doi: 10.1007/s00442-013-2776-7 (PMC3897869; doi:10.1007/s00442-013-2776-7)
Supplement: Supplementary file 2 — Supplementary material 2 (DOC 96 kb) [file 442_2013_2776_MOESM2_ESM.doc]

**Appendix 2** Model selection results from an analysis of the effects of continental origin (continent), mean length at age 2 (L2), air temperature degree-days (temp), and log_e_-transformed surface area of the water body (area) on three life history traits of pumpkinseed populations. Candidate models are ranked by the Akaike Information Criterion corrected for small sample size (AICc), with the differences between models shown as ∆*_i_*. Also shown for each model are the number of parameters (*K*), Akaike weight (w*_i_*), relative likelihood, and proportion of variation explained by the model, adjusted for the number of variables (*R*^2^_adj_). This figure differs from Table 3 in that all tested models are shown here.

| Trait | Independent variable(s) | *K* | AICc | Relative likelihood | ∆*_i_* | w*_i_* | *R*^2^_adj_ |
| --- | --- | --- | --- | --- | --- | --- | --- |
| Age at maturity | continent, L2, area | 5 | 140.78 | 1.0 | 0.00 | 0.48 | 0.57 |
|  | continent, L2 | 4 | 141.87 | 0.58 | 1.11 | 0.27 | 0.56 |
|  | continent, L2, area, temp | 6 | 143.16 | 0.30 | 2.38 | 0.14 | 0.56 |
|  | continent, L2, temp | 5 | 143.79 | 0.22 | 3.01 | 0.11 | 0.55 |
|  | L2, area | 4 | 157.46 | < 0.001 | 16.69 | 0.001 | 0.44 |
|  | L2, area, temp | 5 | 159.79 | < 0.001 | 19.02 | <0.001 | 0.43 |
|  | continent, temp | 4 | 166.33 | < 0.001 | 25.56 | <0.001 | 0.40 |
|  | continent, area, temp | 5 | 167.21 | < 0.001 | 26.44 | <0.001 | 0.40 |
|  | L2 | 3 | 169.34 | < 0.001 | 28.57 | <0.001 | 0.32 |
|  | L2, temp | 4 | 170.69 | < 0.001 | 29.92 | <0.001 | 0.32 |
|  | Area, temp | 4 | 181.07 | < 0.001 | 40.30 | <0.001 | 0.26 |
|  | continent | 3 | 186.43 | < 0.001 | 45.66 | <0.001 | 0.18 |
|  | continent, area | 4 | 186.94 | < 0.001 | 46.16 | <0.001 | 0.19 |
|  | temp | 3 | 187.37 | < 0.001 | 46.60 | <0.001 | 0.17 |
|  | area | 3 | 201.19 | < 0.001 | 60.42 | <0.001 | <0.01 |
| Len at maturity | continent, L2, area | 5 | 558.75 | 1.0 | 0.00 | 0.36 | 0.39 |
|  | continent, L2 | 4 | 558.92 | 0.92 | 0.16 | 0.34 | 0.38 |
|  | continent, L2, temp | 5 | 560.96 | 0.33 | 2.20 | 0.12 | 0.37 |
|  | continent, L2, area temp | 6 | 561.09 | 0.31 | 2.34 | 0.11 | 0.38 |
|  | continent, area | 4 | 563.85 | 0.08 | 5.10 | 0.03 | 0.40 |
|  | continent | 3 | 564.69 | 0.05 | 5.93 | 0.02 | 0.38 |
|  | continent, area, temp | 5 | 566.19 | 0.02 | 7.44 | 0.01 | 0.39 |
|  | continent, temp | 4 | 566.38 | 0.02 | 7.62 | 0.01 | 0.38 |
|  | L2, area | 4 | 576.94 | < 0.001 | 18.19 | <0.001 | 0.18 |
|  | L2, area, temp | 5 | 578.96 | < 0.001 | 20.21 | <0.001 | 0.17 |
|  | area | 3 | 583.11 | < 0.001 | 24.36 | <0.001 | 0.18 |
|  | area, temp | 4 | 585.31 | < 0.001 | 26.56 | <0.001 | 0.17 |
|  | L2 | 3 | 589.28 | < 0.001 | 30.53 | <0.001 | <0.01 |
|  | L2, temp | 4 | 589.65 | < 0.001 | 30.89 | <0.001 | <0.01 |
|  | temp | 3 | 595.61 | < 0.001 | 36.85 | <0.001 | <0.01 |
| GSI | continent, L2 | 4 | 279.62 | 1.0 | 0.00 | 0.42 | 0.40 |
|  | continent | 3 | 281.85 | 0.33 | 2.23 | 0.14 | 0.40 |
|  | continent, L2, temp | 5 | 281.85 | 0.33 | 2.23 | 0.14 | 0.39 |
|  | continent, L2, area | 5 | 281.89 | 0.32 | 2.27 | 0.13 | 0.39 |
|  | continent, temp | 4 | 283.57 | 0.14 | 3.94 | 0.06 | 0.39 |
|  | continent, area | 4 | 283.67 | 0.13 | 4.05 | 0.06 | 0.39 |
|  | continent, L2, area, temp | 6 | 284.16 | 0.10 | 4.53 | 0.04 | 0.38 |
|  | continent, area, temp | 5 | 285.76 | 0.05 | 6.14 | <0.001 | 0.39 |
|  | L2, area | 4 | 308.87 | < 0.001 | 29.25 | <0.001 | 0.07 |
|  | L2, area, temp | 5 | 310.99 | < 0.001 | 31.37 | <0.001 | 0.06 |
|  | area | 3 | 312.47 | < 0.001 | 32.85 | <0.001 | 0.05 |
|  | L2 | 3 | 313.07 | < 0.001 | 33.45 | <0.001 | 0.04 |
|  | area, temp | 4 | 314.11 | < 0.001 | 34.49 | <0.001 | 0.02 |
|  | L2, temp | 4 | 314.22 | < 0.001 | 34.59 | <0.001 | <0.01 |
|  | temp | 3 | 316.83 | < 0.001 | 37.21 | <0.001 | <0.01 |
|  |  |  |  |  |  |  |  |
|  |  |  |  |  |  |  |  |
|  |  |  |  |  |  |  |  |
|  |  |  |  |  |  |  |  |
|  |  |  |  |  |  |  |  |
|  |  |  |  |  |  |  |  |
